# [(2*R*,3*S*,5*R*)-3-Acet­oxy-5-(5-formyl-2,4-dioxo-1,2,3,4-tetra­hydro­pyrimidin-1-yl)-2,3,4,5-tetra­hydro­furan-2-yl]methyl acetate

**DOI:** 10.1107/S1600536811041304

**Published:** 2011-10-12

**Authors:** Xue-Fei Jia, Nan Liu, Liang-Liang Fang, Xin-Ying Zhang

**Affiliations:** a School of Chemistry and Environmental Science, Henan Key Laboratory for Environmental Pollution Control, Henan Normal University, Xinxiang, Henan 453007, People’s Republic of China

## Abstract

In the two independent but very similar mol­ecules (*A* and *B*) of the title compound, C_14_H_16_N_2_O_8_, both six-membered pyrimidine rings are nearly planar [maximum deviations = 0.010 (3) Å in *A* and 0.028 (3) Å in *B*]. The five-membered furan­ose ring in mol­ecule *A* adopts an envelope conformation, while the same ring in mol­ecule *B* has a twisted conformation. In the crystal, the *A* mol­ecules are linked *via* a pair of inter­molecular N—H⋯O hydrogen bonds, forming dimers. Each *A* mol­ecule is further linked to a *B* mol­ecule *via* a second N—H⋯O hydrogen bond. There are also a number of C—H⋯·O inter­actions present, leading to the formation of a three-dimensional network.

## Related literature

For the bioactivity of 5-substituted pyrimidine nucleosides, see: De Clercq (2005 ▶); Agrofoglio *et al.* (2003 ▶); Lee *et al.* (2009 ▶). For the use of the title compound as a synthon for the preparation of a variety of nucleoside derivatives, see: Fan *et al.* (2006*a* ▶,*b* ▶, 2010 ▶, 2011 ▶); Zhang *et al.* (2009 ▶). For related structures of uridines, see: Luo *et al.* (2007 ▶); Low & Wilson (1984 ▶).
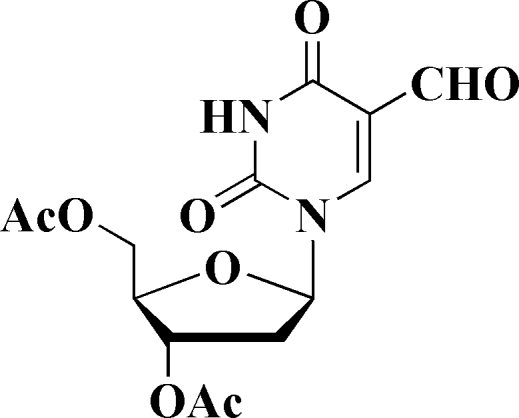


## Experimental

### 

#### Crystal data

C_28_H_32_N_4_O_16_
*M* *_r_* = 680.58
Orthorhombic, 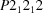

*a* = 15.5268 (18) Å
*b* = 29.977 (4) Å
*c* = 6.6207 (8) Å
*V* = 3081.6 (6) Å^3^
*Z* = 4
Mo *K*α radiation
μ = 0.12 mm^−1^
*T* = 296 K
0.24 × 0.18 × 0.09 mm

#### Data collection

Bruker SMART CCD area-detector diffractometer
Absorption correction: multi-scan (*SADABS*; Bruker, 2007 ▶) *T*_min_ = 0.971, *T*_max_ = 0.989
23677 measured reflections
5719 independent reflections
3519 reflections with *I* > 2σ(*I*)
*R*_int_ = 0.060

#### Refinement

*R*[*F* ^2^ > 2σ(*F* ^2^)] = 0.045
*wR*(*F* ^2^) = 0.110
*S* = 1.01
5719 reflections
437 parameters
H-atom parameters constrained
Δρ_max_ = 0.14 e Å^−3^
Δρ_min_ = −0.17 e Å^−3^
Absolute structure: Flack (1983 ▶), 2320 Friedel pairs
Flack parameter: −0.6 (12)

### 

Data collection: *SMART* (Bruker, 2007 ▶); cell refinement: *SAINT* (Bruker, 2007 ▶); data reduction: *SAINT*; program(s) used to solve structure: *SHELXS97* (Sheldrick, 2008 ▶); program(s) used to refine structure: *SHELXL97* (Sheldrick, 2008 ▶); molecular graphics: *SHELXTL* (Sheldrick, 2008 ▶); software used to prepare material for publication: *SHELXTL*.

## Supplementary Material

Crystal structure: contains datablock(s) I, global. DOI: 10.1107/S1600536811041304/su2320sup1.cif

Structure factors: contains datablock(s) I. DOI: 10.1107/S1600536811041304/su2320Isup2.hkl

Supplementary material file. DOI: 10.1107/S1600536811041304/su2320Isup3.cml

Additional supplementary materials:  crystallographic information; 3D view; checkCIF report

## Figures and Tables

**Table 1:** Hydrogen-bond geometry (Å, °)

*D*—H⋯*A*	*D*—H	H⋯*A*	*D*⋯*A*	*D*—H⋯*A*
N2—H2⋯O6^i^	0.86	1.96	2.817 (3)	172
N4—H4*A*⋯O5	0.86	2.08	2.935 (4)	173
C3—H3⋯O13	0.98	2.57	3.489 (4)	156
C13—H13⋯O2	0.93	2.54	3.402 (4)	155
C16—H16*A*⋯O7^ii^	0.97	2.41	3.312 (4)	155
C18—H18⋯O10^iii^	0.98	2.46	3.257 (4)	138
C21—H21*A*⋯O4^iv^	0.96	2.51	3.441 (5)	162

Symmetry codes: (i) 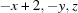; (ii) 
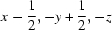
; (iii) 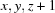; (iv) 
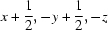
.

**Fig. 1.**
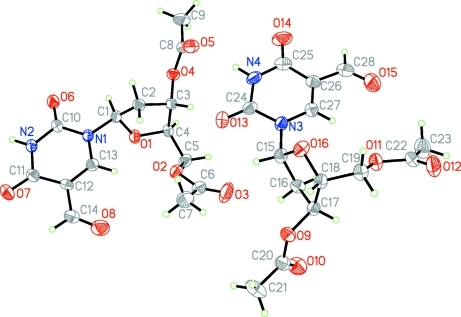
Molecular structure of the two independent molecules (A left; B right) of the title compound, with displacement ellipsoids drawn at the 30% probability level.

**Fig. 2.**
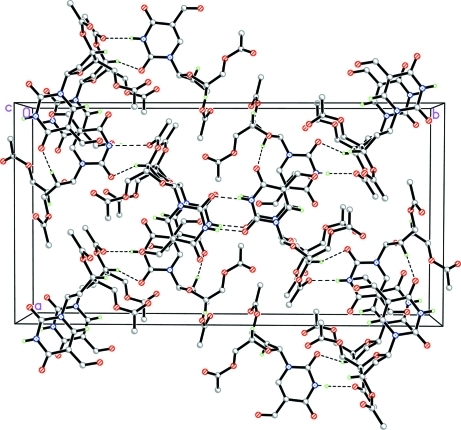
Crystal packing of the title compound, viewed along the c axis. The intermolecular N—H···O hydrogen bonds are shown as dashed lines.

## supplementary crystallographic information

### Comment

Many pyrimidine nucleosides with modification on the 5-position of the pyrimidine ring have drawn much attention due to their interesting pharmacological properties, such as antitumor, antiviral, and antimicrobial activities (De Clercq *et al.*, 2005; Agrofoglio *et al.*, 2003, Lee *et al.*, 2009). The title compound has been used as a powerful synthon for the preparation of a variety of nucleoside derivatives due to the rich and extensive chemistry of the aldehyde carbonyl (Fan *et al.*, 2006*a*, 2006*b*, 2010, 2011; Zhang *et al.*, 2009). However, its crystal structure has not been reported as yet.

The absolute structure of the title compound is known because the synthetic procedure does not affect stereogenic atoms of the starting compound. In the two independent (A & B) but very similar molecules of the title compound (Fig. 1) all the bond lengths and bond angles are within normal ranges. In molecule A the O1—C4 bond is a little longer than bond O1—C1, as is bond O16-C15 compared to bond O16-C18 in molecule B. This is similar to the situation in 2'-deoxy-3',5'-di-*O*-acetyluridine (Luo *et al.*, 2007), but different to that in 2,3,5-triacetyluridine (Low & Wilson, 1984).

The pyrimidine rings in both molecules are planar [maximum deviations being 0.010 (3) Å in A and 0.028 (3) Å in B]. The atoms connected directly with the pyrimidine ring and the atoms in the aldehyde carbonyl group in the 5-position of the pyrimidine ring are coplanar with the pyrimidine ring, which means there is an exstensive conjugated system in each molecule. The five-membered furanose ring in molecule A adopts an envelope conformation with atom C2 at the flap, while in molecule B the five-membered ring is twisted on bond C15-C16.

In the crystal, two A molecules form a pseudosymmetric dimer connected *via* N—H···O hydrogen bonds, involving the N atom of the pyrimidine base and the adjacent carbonyl O atom of the pyrimidine base. Each A molecule is further connected to a B molecule via an N—H···O hydrogen bond involving the N atom of the pyrimidine base and carbonyl O atom of the acetoxy groups in the 3'-position of the furanose ring (see Table 1 and Fig. 2 for details). There are also a number of C-H···O interactions present leading to the formation of a three-dimensional network (Table 1).

### Experimental

The title compound was synthesized following the previously reported procedure (Fan, *et al.*, 2006*b*). Single crystals, suitable for X-ray diffraction analysis, were obtained by slow evaporation of the solvents from a dichloromethane-petroleum ether (1:1 *v*/*v*) solution of the title compound.

### Refinement

The H atoms were positioned geometrically and refined as riding atoms: N—H = 0.86 Å, and C—H = 0.93, 0.98, 0.97, and 0.96 Å for aromatic, methine, methylene, and methyl H atoms, respectively, with *U*_iso_(H) = *x* × *U*_eq_(N,C), where *x* = 1.5 for methyl H atoms, and *x* = 1.2 for all other H atoms. The absolute structure of the title compound is known as the synthetic procedure did not affect the stereogenic atoms of the reactant.

### Crystal data

**Table:**

C_28_H_32_N_4_O_16_	*D*_x_ = 1.467 Mg m^−^^3^
*M**_r_* = 680.58	Mo *K*α radiation, λ = 0.71073 Å
Orthorhombic, *P*2_1_2_1_2	Cell parameters from 2422 reflections
*a* = 15.5268 (18) Å	θ = 2.4–18.6°
*b* = 29.977 (4) Å	µ = 0.12 mm^−^^1^
*c* = 6.6207 (8) Å	*T* = 296 K
*V* = 3081.6 (6) Å^3^	Block, colourless
*Z* = 4	0.24 × 0.18 × 0.09 mm
*F*(000) = 1424	

### Data collection

**Table:**

Bruker SMART CCD area-detector diffractometer	5719 independent reflections
Radiation source: fine-focus sealed tube	3519 reflections with *I* > 2σ(*I*)
graphite	*R*_int_ = 0.060
φ and ω scans	θ_max_ = 25.5°, θ_min_ = 2.4°
Absorption correction: multi-scan (*SADABS*; Bruker, 2007)	*h* = −18→18
*T*_min_ = 0.971, *T*_max_ = 0.989	*k* = −36→36
23677 measured reflections	*l* = −8→8

### Refinement

**Table:**

Refinement on *F*^2^	Secondary atom site location: difference Fourier map
Least-squares matrix: full	Hydrogen site location: inferred from neighbouring sites
*R*[*F*^2^ > 2σ(*F*^2^)] = 0.045	H-atom parameters constrained
*wR*(*F*^2^) = 0.110	*w* = 1/[σ^2^(*F*_o_^2^) + (0.0461*P*)^2^ + 0.0767*P*] where *P* = (*F*_o_^2^ + 2*F*_c_^2^)/3
*S* = 1.01	(Δ/σ)_max_ < 0.001
5719 reflections	Δρ_max_ = 0.14 e Å^−^^3^
437 parameters	Δρ_min_ = −0.17 e Å^−^^3^
0 restraints	Absolute structure: Flack (1983), 2320 Friedel pairs
Primary atom site location: structure-invariant direct methods	Flack parameter: −0.6 (12)

### Special details

**Table:**

Geometry. Bond distances, angles etc. have been calculated using the rounded fractional coordinates. All su's are estimated from the variances of the (full) variance-covariance matrix. The cell esds are taken into account in the estimation of distances, angles and torsion angles
Refinement. Refinement of *F*^2^ against ALL reflections. The weighted *R*-factor *wR* andgoodness of fit *S* are based on *F*^2^, conventional *R*-factors *R* are basedon *F*, with *F* set to zero for negative *F*^2^. The threshold expression of*F*^2^ > σ(*F*^2^) is used only for calculating *R*-factors(gt) *etc*. and isnot relevant to the choice of reflections for refinement. *R*-factors basedon *F*^2^ are statistically about twice as large as those based on *F*, and *R*-factors based on ALL data will be even larger.

### Fractional atomic coordinates and isotropic or equivalent isotropic displacement parameters (Å^2^)

**Table:**

	*x*	*y*	*z*	*U*_iso_*/*U*_eq_	
O1	0.86095 (13)	0.15062 (7)	−0.1052 (3)	0.0481 (8)	
O2	0.92305 (14)	0.23904 (7)	−0.0130 (4)	0.0536 (8)	
O3	0.9059 (2)	0.31223 (8)	−0.0547 (5)	0.0973 (13)	
O4	0.69953 (13)	0.16685 (7)	0.1642 (3)	0.0460 (8)	
O5	0.68476 (16)	0.19298 (8)	0.4790 (4)	0.0634 (9)	
O6	0.91976 (14)	0.03929 (7)	0.0972 (4)	0.0597 (9)	
O7	1.20552 (15)	0.06592 (8)	0.0540 (4)	0.0742 (10)	
O8	1.17118 (16)	0.20098 (9)	0.0293 (5)	0.0822 (11)	
N1	0.96297 (14)	0.11188 (8)	0.0831 (4)	0.0430 (9)	
N2	1.06276 (15)	0.05417 (8)	0.0818 (4)	0.0488 (10)	
C1	0.87385 (18)	0.12800 (9)	0.0809 (5)	0.0428 (11)	
C2	0.85180 (19)	0.16088 (10)	0.2454 (5)	0.0440 (11)	
C3	0.78276 (17)	0.18915 (10)	0.1481 (5)	0.0418 (11)	
C4	0.80634 (19)	0.18894 (10)	−0.0762 (5)	0.0421 (11)	
C5	0.8510 (2)	0.23042 (11)	−0.1460 (5)	0.0540 (12)	
C6	0.9448 (2)	0.28190 (13)	0.0199 (6)	0.0603 (14)	
C7	1.0206 (2)	0.28489 (12)	0.1526 (7)	0.0803 (19)	
C8	0.6580 (2)	0.17068 (11)	0.3399 (6)	0.0467 (11)	
C9	0.5770 (2)	0.14428 (12)	0.3443 (6)	0.0717 (16)	
C10	0.9779 (2)	0.06618 (10)	0.0875 (5)	0.0448 (11)	
C11	1.1334 (2)	0.08179 (11)	0.0667 (5)	0.0493 (12)	
C12	1.11258 (18)	0.12880 (10)	0.0623 (5)	0.0419 (11)	
C13	1.03017 (19)	0.14162 (10)	0.0667 (5)	0.0446 (11)	
C14	1.1828 (2)	0.16136 (13)	0.0510 (5)	0.0550 (12)	
O9	1.01699 (14)	0.43238 (7)	0.3062 (3)	0.0513 (8)	
O10	1.01223 (18)	0.44857 (9)	−0.0227 (4)	0.0786 (11)	
O11	0.78072 (14)	0.48878 (7)	0.5019 (4)	0.0563 (9)	
O12	0.76091 (17)	0.55682 (9)	0.6310 (5)	0.0820 (11)	
O13	0.83321 (16)	0.28502 (7)	0.4225 (4)	0.0648 (10)	
O14	0.54241 (17)	0.29279 (9)	0.4668 (5)	0.0914 (14)	
O15	0.54287 (16)	0.42833 (10)	0.4218 (5)	0.0932 (13)	
O16	0.85943 (14)	0.40460 (7)	0.5944 (3)	0.0543 (8)	
N3	0.77201 (16)	0.35465 (8)	0.4263 (4)	0.0470 (10)	
N4	0.68780 (19)	0.29072 (9)	0.4549 (4)	0.0581 (11)	
C15	0.85591 (19)	0.37705 (10)	0.4219 (5)	0.0488 (11)	
C16	0.8695 (2)	0.40735 (11)	0.2421 (5)	0.0483 (12)	
C17	0.9270 (2)	0.44370 (11)	0.3260 (5)	0.0446 (11)	
C18	0.9070 (2)	0.44455 (10)	0.5522 (5)	0.0444 (11)	
C19	0.8576 (2)	0.48391 (10)	0.6264 (5)	0.0533 (12)	
C20	1.0522 (2)	0.43648 (12)	0.1203 (6)	0.0550 (14)	
C21	1.1458 (2)	0.42467 (15)	0.1230 (7)	0.0840 (18)	
C22	0.7370 (2)	0.52717 (13)	0.5227 (6)	0.0597 (14)	
C23	0.6581 (2)	0.52785 (13)	0.3963 (8)	0.0840 (19)	
C24	0.7698 (2)	0.30800 (11)	0.4335 (5)	0.0503 (12)	
C25	0.6102 (2)	0.31309 (13)	0.4535 (5)	0.0610 (14)	
C26	0.6196 (2)	0.36078 (11)	0.4387 (5)	0.0497 (12)	
C27	0.6985 (2)	0.37920 (11)	0.4317 (5)	0.0477 (12)	
C28	0.5422 (2)	0.38840 (15)	0.4283 (6)	0.0667 (16)	
H1	0.83450	0.10250	0.08870	0.0510*	
H2	1.07330	0.02600	0.08830	0.0590*	
H2A	0.90150	0.17870	0.28230	0.0530*	
H2B	0.83010	0.14580	0.36460	0.0530*	
H3	0.78130	0.21940	0.20390	0.0500*	
H4	0.75370	0.18490	−0.15560	0.0510*	
H5A	0.87120	0.22660	−0.28350	0.0650*	
H5B	0.81130	0.25540	−0.14320	0.0650*	
H7A	1.03510	0.31570	0.17400	0.1210*	
H7B	1.06840	0.26990	0.09040	0.1210*	
H7C	1.00790	0.27110	0.28000	0.1210*	
H9A	0.58980	0.11390	0.37930	0.1080*	
H9B	0.55020	0.14520	0.21360	0.1080*	
H9C	0.53850	0.15670	0.44290	0.1080*	
H13	1.01770	0.17190	0.05840	0.0530*	
H14	1.23920	0.15110	0.06090	0.0660*	
H4A	0.68480	0.26230	0.47120	0.0700*	
H15	0.90210	0.35480	0.42780	0.0590*	
H16A	0.81530	0.41950	0.19440	0.0580*	
H16B	0.89750	0.39160	0.13220	0.0580*	
H17	0.91440	0.47260	0.26310	0.0540*	
H18	0.96160	0.44320	0.62610	0.0530*	
H19A	0.84140	0.47960	0.76650	0.0640*	
H19B	0.89270	0.51060	0.61720	0.0640*	
H21A	1.15370	0.39560	0.06520	0.1270*	
H21B	1.16630	0.42470	0.25980	0.1270*	
H21C	1.17760	0.44620	0.04560	0.1270*	
H23A	0.61210	0.51320	0.46680	0.1260*	
H23B	0.66910	0.51260	0.27130	0.1260*	
H23C	0.64210	0.55820	0.36890	0.1260*	
H27	0.70300	0.41010	0.43060	0.0580*	
H28	0.48900	0.37410	0.42670	0.0800*	

### Atomic displacement parameters (Å^2^)

**Table:**

	*U*^11^	*U*^22^	*U*^33^	*U*^12^	*U*^13^	*U*^23^
O1	0.0548 (13)	0.0426 (12)	0.0468 (14)	0.0117 (10)	0.0016 (12)	0.0004 (11)
O2	0.0525 (14)	0.0384 (13)	0.0699 (16)	−0.0016 (11)	0.0001 (12)	0.0071 (11)
O3	0.106 (2)	0.0449 (15)	0.141 (3)	0.0053 (15)	−0.016 (2)	0.0169 (19)
O4	0.0391 (12)	0.0470 (13)	0.0520 (14)	−0.0058 (11)	0.0035 (11)	−0.0026 (11)
O5	0.0716 (16)	0.0605 (15)	0.0581 (17)	−0.0130 (13)	0.0144 (14)	−0.0046 (14)
O6	0.0457 (13)	0.0382 (12)	0.0953 (19)	0.0004 (11)	0.0056 (14)	0.0012 (13)
O7	0.0418 (14)	0.0747 (17)	0.106 (2)	0.0114 (12)	0.0074 (14)	0.0057 (16)
O8	0.0690 (17)	0.0746 (19)	0.103 (2)	−0.0171 (15)	0.0077 (16)	−0.0053 (18)
N1	0.0351 (14)	0.0342 (14)	0.0596 (18)	0.0014 (12)	−0.0003 (14)	0.0043 (14)
N2	0.0433 (16)	0.0388 (14)	0.0642 (19)	0.0056 (12)	0.0039 (15)	0.0038 (15)
C1	0.0379 (18)	0.0356 (17)	0.055 (2)	0.0017 (14)	−0.0010 (17)	0.0048 (18)
C2	0.0378 (19)	0.050 (2)	0.0441 (19)	−0.0007 (16)	−0.0002 (15)	0.0034 (17)
C3	0.0336 (18)	0.0367 (17)	0.055 (2)	−0.0043 (14)	0.0011 (15)	0.0000 (16)
C4	0.0408 (17)	0.0406 (18)	0.045 (2)	0.0074 (15)	−0.0028 (16)	0.0008 (17)
C5	0.057 (2)	0.051 (2)	0.054 (2)	0.0015 (17)	−0.0032 (18)	0.0143 (17)
C6	0.062 (2)	0.048 (2)	0.071 (3)	−0.0061 (19)	0.012 (2)	0.004 (2)
C7	0.073 (3)	0.064 (3)	0.104 (4)	−0.014 (2)	0.002 (3)	−0.005 (2)
C8	0.045 (2)	0.0421 (19)	0.053 (2)	0.0000 (16)	0.0072 (18)	0.0053 (18)
C9	0.054 (2)	0.082 (3)	0.079 (3)	−0.019 (2)	0.015 (2)	0.002 (2)
C10	0.0403 (19)	0.045 (2)	0.049 (2)	0.0067 (16)	0.0017 (17)	−0.0023 (17)
C11	0.044 (2)	0.058 (2)	0.046 (2)	0.0049 (18)	0.0029 (17)	−0.0002 (18)
C12	0.0345 (17)	0.055 (2)	0.0363 (19)	−0.0038 (15)	0.0058 (15)	−0.0023 (17)
C13	0.048 (2)	0.0408 (17)	0.045 (2)	−0.0033 (16)	−0.0003 (17)	−0.0025 (17)
C14	0.047 (2)	0.061 (2)	0.057 (2)	−0.0062 (18)	0.0075 (18)	−0.006 (2)
O9	0.0400 (13)	0.0585 (15)	0.0554 (15)	0.0020 (11)	−0.0022 (11)	−0.0017 (12)
O10	0.0779 (19)	0.101 (2)	0.0569 (17)	0.0079 (16)	0.0026 (15)	0.0083 (16)
O11	0.0516 (14)	0.0516 (15)	0.0658 (16)	0.0085 (12)	−0.0076 (12)	−0.0083 (12)
O12	0.083 (2)	0.0629 (17)	0.100 (2)	0.0161 (15)	0.0022 (17)	−0.0230 (17)
O13	0.0690 (16)	0.0471 (14)	0.0783 (19)	0.0118 (13)	0.0046 (15)	0.0023 (14)
O14	0.0703 (19)	0.092 (2)	0.112 (3)	−0.0347 (16)	0.0091 (17)	0.0173 (18)
O15	0.0596 (18)	0.092 (2)	0.128 (3)	0.0150 (16)	0.0063 (17)	0.003 (2)
O16	0.0700 (15)	0.0476 (13)	0.0452 (14)	−0.0095 (12)	−0.0084 (13)	0.0002 (11)
N3	0.0451 (16)	0.0400 (16)	0.0560 (18)	−0.0013 (13)	−0.0042 (15)	0.0018 (14)
N4	0.070 (2)	0.0442 (16)	0.060 (2)	−0.0129 (16)	0.0094 (17)	0.0077 (15)
C15	0.0461 (19)	0.0424 (18)	0.058 (2)	−0.0013 (15)	−0.0072 (18)	−0.0066 (19)
C16	0.043 (2)	0.056 (2)	0.046 (2)	−0.0068 (17)	−0.0020 (16)	−0.0090 (17)
C17	0.039 (2)	0.0429 (19)	0.052 (2)	0.0010 (16)	−0.0065 (16)	0.0002 (17)
C18	0.0422 (18)	0.0400 (18)	0.051 (2)	0.0000 (15)	−0.0083 (16)	−0.0029 (17)
C19	0.051 (2)	0.053 (2)	0.056 (2)	0.0004 (17)	−0.0097 (18)	−0.0086 (17)
C20	0.053 (2)	0.055 (2)	0.057 (3)	−0.0045 (18)	0.003 (2)	−0.012 (2)
C21	0.052 (2)	0.123 (4)	0.077 (3)	0.003 (2)	0.010 (2)	−0.029 (3)
C22	0.056 (2)	0.057 (2)	0.066 (3)	0.010 (2)	0.016 (2)	−0.002 (2)
C23	0.061 (3)	0.082 (3)	0.109 (4)	0.019 (2)	−0.014 (3)	−0.005 (3)
C24	0.062 (2)	0.045 (2)	0.044 (2)	−0.0091 (18)	0.0033 (19)	−0.0013 (19)
C25	0.057 (2)	0.076 (3)	0.050 (2)	−0.012 (2)	0.0037 (19)	0.005 (2)
C26	0.046 (2)	0.063 (2)	0.040 (2)	−0.0008 (18)	−0.0004 (17)	0.0042 (18)
C27	0.049 (2)	0.050 (2)	0.044 (2)	0.0031 (17)	−0.0029 (17)	−0.0006 (17)
C28	0.055 (2)	0.088 (3)	0.057 (3)	−0.004 (2)	0.007 (2)	0.008 (3)

### Geometric parameters (Å, °)

**Table:**

O1—C1	1.421 (4)	C12—C13	1.336 (4)
O1—C4	1.441 (4)	C12—C14	1.465 (5)
O2—C5	1.447 (4)	C1—H1	0.9800
O2—C6	1.346 (4)	C2—H2B	0.9700
O3—C6	1.198 (5)	C2—H2A	0.9700
O4—C3	1.459 (3)	C3—H3	0.9800
O4—C8	1.335 (4)	C4—H4	0.9800
O5—C8	1.212 (4)	C5—H5A	0.9700
O6—C10	1.212 (4)	C5—H5B	0.9700
O7—C11	1.220 (4)	C7—H7A	0.9600
O8—C14	1.210 (5)	C7—H7C	0.9600
O9—C20	1.352 (4)	C7—H7B	0.9600
O9—C17	1.444 (4)	C9—H9B	0.9600
O10—C20	1.189 (5)	C9—H9C	0.9600
O11—C22	1.343 (4)	C9—H9A	0.9600
O11—C19	1.458 (4)	C13—H13	0.9300
O12—C22	1.201 (5)	C14—H14	0.9300
O13—C24	1.204 (4)	C15—C16	1.512 (5)
O14—C25	1.219 (4)	C16—C17	1.514 (5)
O15—C28	1.198 (5)	C17—C18	1.530 (5)
O16—C18	1.435 (4)	C18—C19	1.491 (4)
O16—C15	1.411 (4)	C20—C21	1.496 (4)
N1—C10	1.390 (4)	C22—C23	1.484 (5)
N1—C13	1.377 (4)	C25—C26	1.440 (5)
N1—C1	1.466 (4)	C26—C27	1.345 (4)
N2—C10	1.366 (4)	C26—C28	1.461 (5)
N2—C11	1.378 (4)	C15—H15	0.9800
N2—H2	0.8600	C16—H16A	0.9700
N3—C15	1.466 (4)	C16—H16B	0.9700
N3—C24	1.400 (4)	C17—H17	0.9800
N3—C27	1.359 (4)	C18—H18	0.9800
N4—C24	1.382 (4)	C19—H19A	0.9700
N4—C25	1.379 (4)	C19—H19B	0.9700
N4—H4A	0.8600	C21—H21A	0.9600
C1—C2	1.508 (4)	C21—H21B	0.9600
C2—C3	1.511 (4)	C21—H21C	0.9600
C3—C4	1.530 (5)	C23—H23A	0.9600
C4—C5	1.497 (4)	C23—H23B	0.9600
C6—C7	1.471 (5)	C23—H23C	0.9600
C8—C9	1.486 (5)	C27—H27	0.9300
C11—C12	1.446 (4)	C28—H28	0.9300
			
C1—O1—C4	110.4 (2)	H9A—C9—H9B	110.00
C5—O2—C6	117.6 (3)	H9A—C9—H9C	109.00
C3—O4—C8	116.9 (2)	H9B—C9—H9C	109.00
C17—O9—C20	116.9 (2)	C8—C9—H9A	110.00
C19—O11—C22	116.2 (3)	C8—C9—H9B	109.00
C15—O16—C18	110.6 (2)	N1—C13—H13	119.00
C10—N1—C13	120.9 (2)	C12—C13—H13	119.00
C1—N1—C10	118.9 (2)	O8—C14—H14	118.00
C1—N1—C13	120.1 (2)	C12—C14—H14	118.00
C10—N2—C11	127.7 (3)	O16—C15—C16	106.3 (2)
C11—N2—H2	116.00	N3—C15—C16	114.5 (3)
C10—N2—H2	116.00	O16—C15—N3	106.7 (2)
C24—N3—C27	121.3 (3)	C15—C16—C17	103.1 (3)
C15—N3—C24	118.7 (2)	O9—C17—C18	106.8 (2)
C15—N3—C27	119.9 (2)	O9—C17—C16	111.6 (3)
C24—N4—C25	128.5 (3)	C16—C17—C18	104.6 (3)
C24—N4—H4A	116.00	O16—C18—C19	109.4 (2)
C25—N4—H4A	116.00	C17—C18—C19	116.1 (3)
O1—C1—N1	107.4 (2)	O16—C18—C17	106.3 (2)
N1—C1—C2	115.0 (2)	O11—C19—C18	108.3 (3)
O1—C1—C2	106.4 (2)	O9—C20—C21	111.1 (3)
C1—C2—C3	102.7 (3)	O9—C20—O10	122.8 (3)
O4—C3—C4	106.3 (2)	O10—C20—C21	126.1 (4)
C2—C3—C4	104.0 (2)	O12—C22—C23	125.6 (3)
O4—C3—C2	109.9 (2)	O11—C22—C23	111.8 (3)
O1—C4—C3	105.9 (2)	O11—C22—O12	122.6 (3)
O1—C4—C5	110.4 (2)	O13—C24—N3	123.3 (3)
C3—C4—C5	114.0 (3)	O13—C24—N4	123.0 (3)
O2—C5—C4	108.6 (3)	N3—C24—N4	113.6 (3)
O2—C6—C7	110.8 (3)	O14—C25—N4	120.7 (3)
O2—C6—O3	122.1 (3)	N4—C25—C26	113.2 (3)
O3—C6—C7	127.1 (4)	O14—C25—C26	126.0 (3)
O5—C8—C9	124.7 (3)	C25—C26—C28	118.8 (3)
O4—C8—O5	123.0 (3)	C25—C26—C27	120.2 (3)
O4—C8—C9	112.3 (3)	C27—C26—C28	121.0 (3)
N1—C10—N2	114.8 (3)	N3—C27—C26	123.0 (3)
O6—C10—N2	123.0 (3)	O15—C28—C26	124.1 (3)
O6—C10—N1	122.2 (3)	O16—C15—H15	110.00
N2—C11—C12	114.2 (3)	N3—C15—H15	110.00
O7—C11—N2	120.1 (3)	C16—C15—H15	110.00
O7—C11—C12	125.7 (3)	C15—C16—H16A	111.00
C13—C12—C14	121.5 (3)	C15—C16—H16B	111.00
C11—C12—C14	118.9 (3)	C17—C16—H16A	111.00
C11—C12—C13	119.6 (3)	C17—C16—H16B	111.00
N1—C13—C12	122.8 (3)	H16A—C16—H16B	109.00
O8—C14—C12	123.3 (3)	O9—C17—H17	111.00
N1—C1—H1	109.00	C16—C17—H17	111.00
O1—C1—H1	109.00	C18—C17—H17	111.00
C2—C1—H1	109.00	O16—C18—H18	108.00
H2A—C2—H2B	109.00	C17—C18—H18	108.00
C3—C2—H2B	111.00	C19—C18—H18	108.00
C3—C2—H2A	111.00	O11—C19—H19A	110.00
C1—C2—H2B	111.00	O11—C19—H19B	110.00
C1—C2—H2A	111.00	C18—C19—H19A	110.00
O4—C3—H3	112.00	C18—C19—H19B	110.00
C4—C3—H3	112.00	H19A—C19—H19B	108.00
C2—C3—H3	112.00	C20—C21—H21A	110.00
C5—C4—H4	109.00	C20—C21—H21B	109.00
O1—C4—H4	109.00	C20—C21—H21C	110.00
C3—C4—H4	109.00	H21A—C21—H21B	110.00
O2—C5—H5B	110.00	H21A—C21—H21C	109.00
O2—C5—H5A	110.00	H21B—C21—H21C	109.00
H5A—C5—H5B	108.00	C22—C23—H23A	110.00
C4—C5—H5A	110.00	C22—C23—H23B	109.00
C4—C5—H5B	110.00	C22—C23—H23C	109.00
C6—C7—H7B	109.00	H23A—C23—H23B	109.00
C6—C7—H7C	110.00	H23A—C23—H23C	109.00
H7A—C7—H7B	109.00	H23B—C23—H23C	110.00
H7A—C7—H7C	109.00	N3—C27—H27	118.00
H7B—C7—H7C	110.00	C26—C27—H27	119.00
C6—C7—H7A	110.00	O15—C28—H28	118.00
C8—C9—H9C	109.00	C26—C28—H28	118.00
			
C4—O1—C1—N1	−142.9 (2)	C24—N3—C15—O16	−120.1 (3)
C4—O1—C1—C2	−19.2 (3)	C27—N3—C15—O16	57.3 (4)
C1—O1—C4—C5	122.9 (3)	C24—N3—C15—C16	122.7 (3)
C1—O1—C4—C3	−1.0 (3)	C24—N4—C25—C26	−3.8 (5)
C5—O2—C6—C7	−178.2 (3)	C24—N4—C25—O14	177.2 (3)
C6—O2—C5—C4	−147.9 (3)	C25—N4—C24—N3	5.6 (5)
C5—O2—C6—O3	1.0 (5)	C25—N4—C24—O13	−174.5 (3)
C8—O4—C3—C4	−167.3 (2)	O1—C1—C2—C3	31.3 (3)
C3—O4—C8—O5	3.2 (4)	N1—C1—C2—C3	150.1 (2)
C8—O4—C3—C2	80.8 (3)	C1—C2—C3—C4	−31.0 (3)
C3—O4—C8—C9	−176.2 (3)	C1—C2—C3—O4	82.5 (3)
C17—O9—C20—C21	178.7 (3)	C2—C3—C4—C5	−101.1 (3)
C20—O9—C17—C18	−170.0 (3)	O4—C3—C4—O1	−95.5 (2)
C17—O9—C20—O10	−0.3 (5)	C2—C3—C4—O1	20.5 (3)
C20—O9—C17—C16	76.3 (3)	O4—C3—C4—C5	142.9 (2)
C19—O11—C22—O12	2.9 (5)	C3—C4—C5—O2	51.7 (3)
C22—O11—C19—C18	−168.1 (3)	O1—C4—C5—O2	−67.3 (3)
C19—O11—C22—C23	−177.5 (3)	O7—C11—C12—C13	176.4 (3)
C18—O16—C15—N3	−146.0 (2)	O7—C11—C12—C14	−3.0 (5)
C18—O16—C15—C16	−23.4 (3)	N2—C11—C12—C14	178.7 (3)
C15—O16—C18—C19	132.3 (3)	N2—C11—C12—C13	−2.0 (5)
C15—O16—C18—C17	6.1 (3)	C13—C12—C14—O8	−5.9 (5)
C10—N1—C1—O1	−116.1 (3)	C11—C12—C13—N1	2.5 (5)
C13—N1—C1—C2	−58.8 (4)	C11—C12—C14—O8	173.5 (3)
C13—N1—C10—N2	2.3 (4)	C14—C12—C13—N1	−178.1 (3)
C10—N1—C1—C2	125.7 (3)	O16—C15—C16—C17	30.7 (3)
C13—N1—C10—O6	−178.3 (3)	N3—C15—C16—C17	148.1 (3)
C1—N1—C10—N2	177.7 (3)	C15—C16—C17—O9	88.9 (3)
C1—N1—C13—C12	−178.1 (3)	C15—C16—C17—C18	−26.2 (3)
C1—N1—C10—O6	−2.8 (5)	C16—C17—C18—O16	13.4 (3)
C10—N1—C13—C12	−2.7 (5)	O9—C17—C18—O16	−105.0 (3)
C13—N1—C1—O1	59.4 (3)	C16—C17—C18—C19	−108.6 (3)
C11—N2—C10—N1	−2.0 (5)	O9—C17—C18—C19	133.0 (3)
C11—N2—C10—O6	178.6 (3)	C17—C18—C19—O11	52.1 (3)
C10—N2—C11—O7	−176.6 (3)	O16—C18—C19—O11	−68.2 (3)
C10—N2—C11—C12	1.8 (5)	O14—C25—C26—C27	177.7 (4)
C15—N3—C24—N4	174.7 (3)	N4—C25—C26—C28	177.8 (3)
C15—N3—C24—O13	−5.2 (5)	O14—C25—C26—C28	−3.2 (5)
C27—N3—C24—N4	−2.6 (4)	N4—C25—C26—C27	−1.2 (5)
C27—N3—C24—O13	177.5 (3)	C25—C26—C27—N3	3.9 (5)
C24—N3—C27—C26	−1.8 (5)	C27—C26—C28—O15	−3.3 (6)
C27—N3—C15—C16	−60.0 (4)	C28—C26—C27—N3	−175.1 (3)
C15—N3—C27—C26	−179.1 (3)	C25—C26—C28—O15	177.7 (4)

### Hydrogen-bond geometry (Å, °)

**Table:**

*D*—H···*A*	*D*—H	H···*A*	*D*···*A*	*D*—H···*A*
N2—H2···O6^i^	0.86	1.96	2.817 (3)	172
N4—H4A···O5	0.86	2.08	2.935 (4)	173
C3—H3···O13	0.98	2.57	3.489 (4)	156
C13—H13···O2	0.93	2.54	3.402 (4)	155
C16—H16A···O7^ii^	0.97	2.41	3.312 (4)	155
C18—H18···O10^iii^	0.98	2.46	3.257 (4)	138
C21—H21A···O4^iv^	0.96	2.51	3.441 (5)	162

Symmetry codes: (i) −*x*+2, −*y*, *z*; (ii) *x*−1/2, −*y*+1/2, −*z*; (iii) *x*, *y*, *z*+1; (iv) *x*+1/2, −*y*+1/2, −*z*.
